# An exploratory causal analysis of the relationships between the brain age gap and cardiovascular risk factors

**DOI:** 10.3389/fnagi.2022.941864

**Published:** 2022-08-22

**Authors:** Pauline Mouches, Matthias Wilms, Jordan J. Bannister, Agampreet Aulakh, Sönke Langner, Nils D. Forkert

**Affiliations:** ^1^Biomedical Engineering Program, University of Calgary, Calgary, AB, Canada; ^2^Hotchkiss Brain Institute, University of Calgary, Calgary, AB, Canada; ^3^Department of Radiology, University of Calgary, Calgary, AB, Canada; ^4^Alberta Children’s Hospital Research Institute, University of Calgary, Calgary, AB, Canada; ^5^Schulich School of Engineering, University of Calgary, Calgary, AB, Canada; ^6^Institute for Diagnostic Radiology and Neuroradiology, Rostock University Medical Center, Rostock, Germany

**Keywords:** cardiovascular risk factors, brain aging (normal), brain age gap, causal analyses, Bayesian network

## Abstract

The brain age gap (BAG) has been shown to capture accelerated brain aging patterns and might serve as a biomarker for several neurological diseases. Moreover, it was also shown that it captures other biological information related to modifiable cardiovascular risk factors. Previous studies have explored statistical relationships between the BAG and cardiovascular risk factors. However, none of those studies explored causal relationships between the BAG and cardiovascular risk factors. In this work, we employ causal structure discovery techniques and define a Bayesian network to model the assumed causal relationships between the BAG, estimated using morphometric T1-weighted magnetic resonance imaging brain features from 2025 adults, and several cardiovascular risk factors. This setup allows us to not only assess observed conditional probability distributions of the BAG given cardiovascular risk factors, but also to isolate the causal effect of each cardiovascular risk factor on BAG using causal inference. Results demonstrate the feasibility of the proposed causal analysis approach by illustrating intuitive causal relationships between variables. For example, body-mass-index, waist-to-hip ratio, smoking, and alcohol consumption were found to impact the BAG, with the greatest impact for obesity markers resulting in higher chances of developing accelerated brain aging. Moreover, the findings show that causal effects differ from correlational effects, demonstrating the importance of accounting for variable relationships and confounders when evaluating the information captured by a biomarker. Our work demonstrates the feasibility and advantages of using causal analyses instead of purely correlation-based and univariate statistical analyses in the context of brain aging and related problems.

## Introduction

The so-called brain age gap (BAG) has been recently proposed as an imaging-derived early biomarker for several neurodegenerative diseases (Baecker et al., 2021). Estimating the BAG can be performed non-invasively and without ionizing radiation, since it is usually based on magnetic resonance imaging (MRI). This biomarker can capture abnormal brain aging (e.g., accelerated brain atrophy) by calculating the difference between the biological brain age, typically estimated from MRI data using machine learning models, and the chronological age. Within this context, it potentially allows a wide-spread application for the early detection of dementia and psychological disorders (Franke and Gaser, 2019).

In addition to being a reliable biomarker for neurodegenerative diseases such as Alzheimer’s, it has also been reported that the BAG is correlated with clinical and life behavioral factors that are assumed to be related to early aging. For example, cardiovascular risk factors such as elevated blood pressure, waist-to-hip ratio, and cigarette smoking (Cole, 2020; de Lange et al., 2020; Beck et al., 2021) have been associated with an increased BAG. So far, these associations have typically been analyzed independently for each specific factor in a univariate way, sometimes adjusting for the effects of age, sex, or image acquisition parameters (Cole, 2020; de Lange et al., 2020). However, such confounders are frequently determined only based on intuition and employed confounding variable adjustment schemes do not provide any information about causal relationships. Moreover, it is well known that, for example, cardiovascular risk factors interact with each other (e.g., body-mass-index, waist-to-hip ratio, and blood pressure), which is often ignored in standard univariate correlational analyses. We, therefore, argue that there is currently a lack of models/studies that systematically investigate causal relationships in brain aging.

In this work, we aim to (1) discover potential relationships between cardiovascular risk factors and the BAG, (2) build a mathematical model representing these interactions, and (3) use this model to analyze these interactions by performing causal inference.

Causal structure learning/discovery algorithms allow to discover dependencies between variables from observational data, and generate a directed acyclic graph representing the relationships between these variables (Heinze-Deml et al., 2018). In the graph, each variable of interest is a node and (causal) interactions are represented through directed edges between nodes. Once the directed acyclic graph is known, Bayesian networks can be used to model conditional probabilities. Bayesian networks are probabilistic graphical models representing the joint probability distribution of a set of random variables, where interactions between random variables are defined through a directed acyclic graph. The Bayesian network can be used to assess probability distributions on the data using Bayesian inference (Larrañaga and Moral, 2011). Indeed, the probability distribution of a variable of interest given other known variables can be observed within the data and used to infer the values of the variable of interest on new data. In the field of neuroimaging, Bayesian networks have been used as predictive tools, for example, for post-stroke outcome (Park et al., 2018), or as tools to understand the mechanisms of a pathology and identify reliable biomarkers, such as in mild cognitive impairment and Alzheimer’s diseases (Jin et al., 2016; Khanna et al., 2018).

Beyond Bayesian inference, which allows to evaluate conditional probabilities on observational data, causal inference allows to evaluate the effect of an independent variable on another. Therefore, causal inference allows us to alter the value of one variable of the Bayesian network (i.e., to intervene on the network) and to assess its causal effects on other variables (Pearl, 2012). This property is especially interesting in the context of cardiovascular risk factors, as these are often modifiable. Therefore, if a factor (e.g., smoking) is found to cause accelerated brain aging, a specific change in lifestyle (e.g., quit smoking) could help preventing negative effects. For these reasons, Bayesian networks are well-suited to investigate the causal relations between the brain age gap and cardiovascular risk factors and, to the best of our knowledge, have not been used in this context yet.

In this work, we used cross-sectional T1-weighted MRI datasets and cardiovascular risk factors from 2025 adults. First, brain tissue features were extracted from the T1-weighted images, which were used to predict the biological brain age using a trained multilayer perceptron regression model. The BAG was then computed by calculating the difference between the chronological and biological brain age. A cross-validation scheme was employed to maximize the data available while still allowing to train robust models. Then, a causal structure learning/discovery algorithm was used to determine a directed acyclic graph representing the relationships between these variables. The graph was further used to fit conditional probabilities to create a Bayesian network, which gives a factorized probabilistic description of the relationships between cardiovascular risk factors and the BAG. Finally, causal inference was used to evaluate the causal effect of each cardiovascular risk factors on the BAG. The use of Bayesian networks as a novel approach to study the BAG allows us to (1) identify causal relationships between variables, (2) observe conditional probability distributions of the variables, and (3) intervene on the model to isolate the causal effect of each variable on the BAG.

## Materials and methods

### Clinical data

Cross-sectional T1-weighted MRI datasets and cardiovascular risk factors data from the Study of Health in Pomerania (SHIP) were used in this secondary study. The goal of the SHIP study was to gather general population data by randomly selecting participants within the region of Pomerania, Germany (Völzke et al., 2011). The data sample used in this secondary study includes 2025 participants (21–82 years old; mean: 51 ± 14) with no known pathologies in brain MRI scans. T1-weighted MRI data acquisition was performed using a single 1.5T MRI scanner (Magnetom Avanto; Siemens Medical Solutions, Erlangen, Germany) and the following acquisition parameters: TR = 1,900 ms, TE = 3.4 ms, flip angle = 15°, spacing = 1.0 mm^3^ × 1.0 mm^3^ × 1.0 mm^3^. Cardiovascular risk factors were assessed in a single session, at the time of imaging data acquisition. The cardiovascular risk factors available for this work include body-mass-index (BMI) [kg/m^2^], waist-to-hip ratio (WHR), systolic blood pressure (BP) [mmHg], smoking history (encoding the following information: smoker versus non-smoker; past versus current smoker; regular versus occasional smoker), and alcohol consumption (number of glasses of alcohol per week) (see Table 1).

**TABLE 1:** Demographics and cardiovascular risk factors.

	Mean (standard deviation)
Sex	F: 1050; M: 975
Age	F: 50.9 (13.5); M: 50.6 (14.3)
Body-mass-index	F: 26.90 (4.65); M: 28.02 (3.70)
Systolic blood pressure	F: 120.6 (15.6); M: 133.6 (14.9)
Waist-to-hip-ratio	F: 0.82 (0.062); M: 0.94 (0.07)
Smoking (0: non-smoker; 1: past smoker; 2: current smoker) (males%)	0: 803 (38%); 1: 729 (57%); 2: 493 (51%)
Alcohol drinking (0: non-drinker; 1: ≤1 glass/week; 1: >1 glass/week) (males%)	0: 150 (40%); 1: 547 (24%); 2: 1328 (59%)

All participants provided written informed consent and the SHIP study was approved by the local ethics commission of the University of Greifswald (BB 39/08, 19.06.2008).

### Brain age prediction

Morphometric features, including subcortical and cortical structure volume, surface area, and cortical thickness, were computed based on the T1-weighted MRI data using Fastsurfer (Henschel et al., 2020). Briefly, Fastsurfer replicates the Freesurfer pipeline (Fischl et al., 2002) but allows faster computation by utilizing deep learning methods. Fastsurfer first uses a deep learning-based model to segment 95 anatomical structures, and then extracts morphological measurements through surface reconstruction. Using this approach, 223 features were extracted for each subject. All segmentation masks resulting from the Fastsurfer pipeline were visually inspected to ensure accurate segmentation. Datasets with suboptimal segmentation results were not included in this work.

Brain age prediction was conducted using a multi-layer perceptron (MLP) that uses the morphological features extracted from the neuroimaging data as inputs. The MLP architecture was optimized using 10-fold cross validation. MLP architectures with one to four hidden layers, with the following number of neurons per layer were tested: (256); (256, 128); (256, 128, 64); (256, 128, 64, 32). The best performing architecture as defined by the lowest mean absolute error (MAE) when comparing the chronological and predicted age for the test data was then used to predict the brain age of each subject within a 10-folds cross validation approach. Within each cross-validation iteration, nine folds were used for training and one fold was used for testing. Within the data from the nine training folds, 15% of the data were assigned to the validation set used to determine the optimal number of training epochs. All data splits were performed in an age stratified fashion.

Next, estimated brain age predictions were adjusted for age-related bias, following (de Lange et al., 2020). It is well known that brain age prediction models tend to overestimate the age of younger subjects and underestimate the age of elderly subjects. A likely reason for this is the regression toward the mean phenomenon (Liang et al., 2019). To adjust for age-related bias, the slope (α) and intercept (β) of the regression line for the chronological vs. predicted age relation was estimated in each cross-validation iteration using the validation data as follows:


PredictedAge=α×ChronologicalAge+β

Then, correction was applied on the test data:


CorrectedPredictedAge=PredictedAge+[ChronologicalAge-(ChronologicalAge×α+β)]

### Bayesian network

A Bayesian network is defined as a directed acyclic graph with a set of edges and nodes representing random variables *X* = {*X*_1_,*X*_2_,…,*X*_*n*_}. In a Bayesian network, the joint probability distribution is defined in a factorized way as:


$$P\,\left(X\right)=\prod_{i=1}^{n}P\,\left(X_{i}|\text{parents}\,\left(X_{i}\right)\right),$$

where *parents*(*X*_*i*_) are *X*_*i*_’s parent nodes in the graph. In this work, a Bayesian network was learned using the following random variables: age, sex, five cardiovascular risk factors (BMI, WHR, BP, smoking and alcohol drinking, as described in Table 1), and the age-adjusted BAG (estimated brain age – chronological age). The CausalNex package (Welcome to CausalNex’s API, 2020) was used for the model learning, validation, and evaluation steps described in the following.

First, the structure of the directed acyclic graph was automatically learned using the NO TEARS algorithm (Zheng et al., 2018) implementation from the CausalNex package (Welcome to CausalNex’s API, 2020). NO TEARS is a score-based, state-of-the-art structure learning algorithm that has been successfully used for other healthcare related problems (Gencoglu and Gruber, 2020; Marchezini et al., 2022). A list of forbidden edges was given to the algorithm as constraints:

1. None of the variables can affect age and sex;
2. BAG cannot affect any of the variables;
3. BMI, WHR, and BP cannot affect smoking and alcohol drinking status.

Structure learning additionally requires a threshold to discard the weaker edges from the structure. This threshold was determined at the model validation step (see below).

Once the structure is learned, the conditional probability distributions of the Bayesian network can be fitted. To do so, the CausalNex package required the variables to be discretized. Therefore, the following thresholds were chosen, following common categorization schemes (Ashwell, 2011; Mancia et al., 2014), for each cardiovascular risk factor: BMI: [<25, 25–30, >30]; BP: [<120, 120–140, >140]; Males WHR: [<0.95, 0.95–1, >1]; Females WHR: [<0.8, 0.8–0.85, >0.85]; Age: [<35, 35–65, >65]. Smoking and alcohol drinking were categorized as described in Table 1. The brain age gap was categorized as [<–3, –3 to 3, >3], to generate three balanced classes representing delayed brain aging, normal brain aging, and accelerated brain aging, respectively. As all cardiovascular risk factors included in the model were split into three categories, we further refer to them as “low,” “normal” and “high” in the following descriptions. Discretization of the continuous cardiovascular risk factors resulted in the following percentage of males in each category: BMI: [low: 33%, normal: 58%, high: 51%], BP: [low: 25%, normal: 56%, high: 72%]; WHR: [low: 58%, normal: 37%, high: 65%]; Age: [low: 53%, normal: 47%, high: 50%]. Finally, Bayesian parameter estimation was used to fit the probabilities.

The model was validated by computing the area under the receiver operating characteristics curve (AUC) while performing classification for each node of the directed acyclic graph using Bayesian inference on the test data. Therefore, for the random variable *X*_*1*_, *P*(*X*_1_|*X*_2_,…,*X*_*n*_) was computed to determine the most probable value of *X*_*1*_ given all other variables. 10-fold cross validation was used, whereas in each iteration, nine folds were used to fit the conditional probabilities and one fold to test the model. The 10-fold cross-validation was repeated 10 times to ensure robustness of the results, and the average AUCs are reported, for each node and for the whole model. The evaluation stage was used to (1) ensure the model’s quality and (2) determine the optimal threshold to discard the weakest edges of the directed acyclic graph. Therefore, the threshold was chosen to maximize both, the average AUC over all the graph nodes and the BAG node AUC. After evaluating the model using the cross-validation approach, the full database was used to learn the final Bayesian network on which the observation and intervention experiments were performed as described below.

First, the conditional distributions from the trained model were analyzed to learn more about the interactions directly observable from the data. To do so, the probability distribution of each node, conditioned on the value of one of its parent nodes while marginalizing over the remaining ones, was evaluated. Therefore, if *X*_*2*_ is a parent of *X*_*1*_, computing *P*(*X*_1_|*X*_2_ = *x*_2_) answers the question “What is the probability distribution of *X*_*1*_ given that we observe that *X*_*2*_ = *x*_*2*_?”. For instance, we can evaluate the probability of having a high BAG given that someone is smoking.

Then, interventions were used to evaluate causal inferences. Such interventions, for instance, could simulate a world in which some treatment of lifestyle changes would occur. Therefore, interventions simulate conditions that would not be applicable in the real world (such as a world in which everyone smokes). Do-Calculus was used to perform causal inference after intervening on the network (Pearl, 2012). The *do*() operator indicates an intervention on one or several nodes of the directed acyclic graph by fixing the value of the corresponding random variable. *P*(*X*_1_|*do*(*X*_2_ = *x*_2_)) answers the following question: “What would the probability distribution of *X*_*1*_ be if we would intervene on the model and set *X*_*2*_ = *x*_*2*_?”. Thus, parents’ nodes of *X*_*2*_ do not influence it anymore, and the confounding bias free effects of *X*_*2*_ on *X*_*1*_ can be determined. For instance, let us assume that sex is a parent node of smoking and both sex and smoking are parents of the BAG node. Intervening on smoking to evaluate its causal effect on the BAG would imply ignoring the causal influence of sex on smoking, as intervening means manually fixing the value. Do-Calculus allows to transform an expression containing the *do*() operator into an expression without the *do*() operator, which can be computed from the observational data. In this work, intervention was applied on the parents’ nodes of the BAG node, individually, to evaluate the confounding bias free effect of each isolated risk factor on the BAG. Intervention consisted of setting the distribution of each risk factor category (low, normal, high) to 100% and the other categories to 0% and evaluating the probability distribution of the BAG. Identifying risk factors causing the most accelerated brain aging can help evaluating the lifestyle changes required to reduce accelerated brain aging.

## Results

The best performing brain age prediction model architecture had two hidden layers with 256 and 128 neurons, respectively. Brain age prediction resulted in an average MAE of 5.2 years comparing the predicted and chronological age over the 10 folds. After age-bias correction, the average cross-validation MAE decreased to 4.8 years. These results are comparable to the ones observed in other studies using features extracted with Freesurfer (Beck et al., 2021; Lombardi et al., 2021).

**FIGURE 1:**
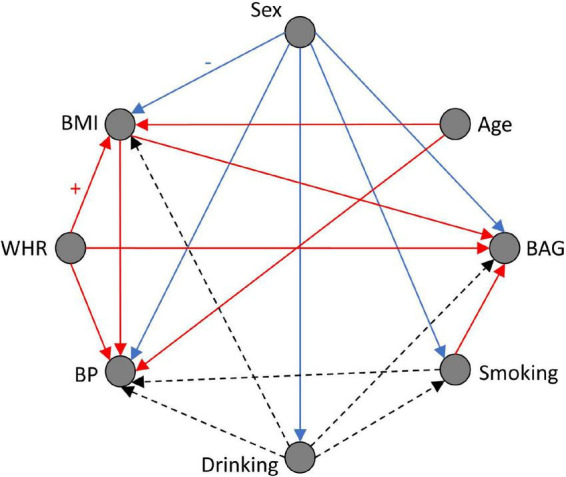
Bayesian network structure (directed acyclic graph), and conditional probability distribution visualization. Red edge: positive association. Blue edge: negative association. Dashed edge: non-linear association. Sex: Male = 0, Female = 1; BAG, brain age gap; BMI, body mass index; BP, blood pressure; WHR, waist-to-hip ratio.

Figure 1 shows the learned Bayesian network’s directed acyclic graph resulting from the analysis of the data available for this work. The graph shows directed edges between the nodes and the colors represent the directionality of the relationship between each node and its children, evaluated by observing conditional probabilities. Red edges indicate positive associations: when the parent node value is high, the child node value is more likely to be high. Blue edges indicate negative associations: when the parent node value is high, the child node value is more likely to be low. Therefore, Figure 1 shows intuitive results such as BMI and BP being more likely to be high when WHR is high, BAG being more likely to be high (i.e., accelerated brain aging) when WHR, BMI, and smoking are high. Males (sex = 0) are more likely to have higher BMI, BP, and to drink and to smoke more. Dashed edges represent non-linear associations. The results suggest that non-drinkers and regular-drinkers are more likely to have a high BAG, BP, or BMI compared to occasional drinkers. Overall, these results support the validity of the graph given current knowledge.

Results of the model validation are reported in Table 2. The average AUC over all the nodes and the repeated 10-folds cross-validation is 0.629. All AUC values are above chance level (>0.5), with the lowest AUC reported for the BAG (0.539), and the highest for sex (0.757).

**TABLE 2:** Area under the ROC curve (AUC) for all model nodes.

	BAG	Age	Sex	BP	BMI	WHR	Smoking	Drinking	Average of all nodes
AUC CV average (CV std)	0.539 (0.0034)	0.588 (0.0037)	0.757 (0.0029)	0.664 (0.0030)	0.688 (0.0023)	0.660 (0.0035)	0.546 (0.0038)	0.593 (0.0038)	0.629 (0.0017)

The AUC average and standard deviation (in brackets) are reported across the ten times repeated 10-fold cross validation (CV).

BAG, brain age gap; BMI, body mass index; BP, blood pressure; WHR, waist-to-hip ratio.

**FIGURE 2:**
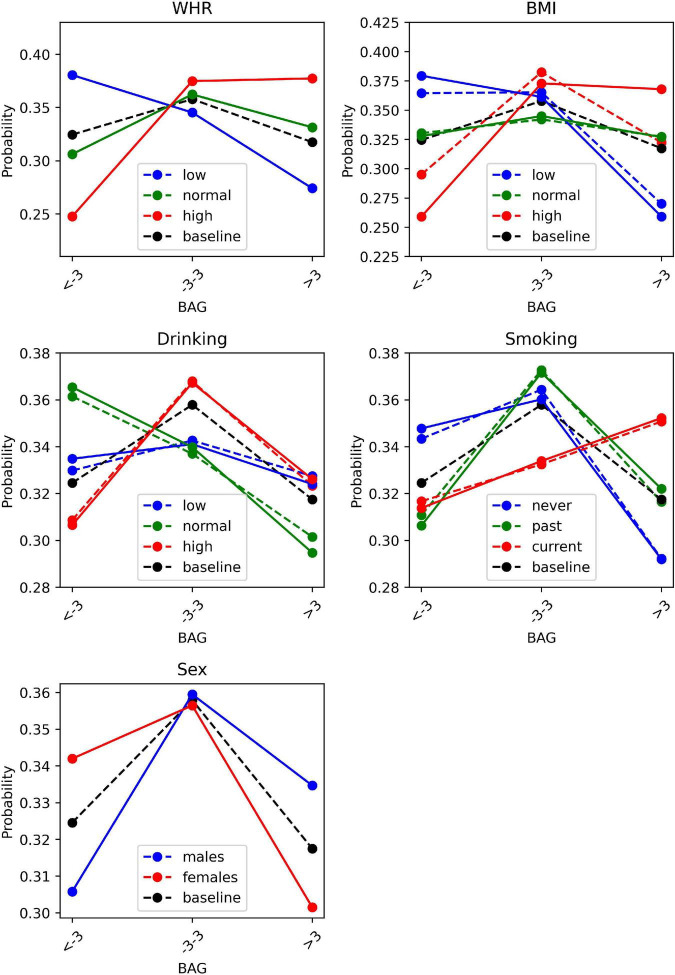
BAG distributions when observing (plain lines) or intervening (dashed lines) on the cardiovascular risk factors. BAG, brain age gap; BMI, body mass index; WHR, waist-to-hip ratio.

Figure 2 illustrates the conditional probability distribution of the BAG when observing (plain lines) and intervening (dashed lines) on the Bayesian network. For nodes without parents in the graph (WHR and sex), observing and intervening leads to the same results. For nodes that have parents that also impact the BAG (BMI, smoking and drinking), observing and intervening differs. Observing while conditioning on parent nodes is equivalent to narrow the focus. Intervening means modifying the model by manually fixing a variable value (Glymour et al., 2016).

As expected, Figure 2 suggests that the causal effects of BMI (dashed line: intervention) on the BAG are generally different than the observed correlational effects of BMI (plain line: observation). For instance, the observational distribution *P*(*BAG*|*BMI* = *high*) is: {low BAG: 0.26, normal BAG: 0.37, high BAG: 0.37} (plain red line), while the interventional distribution *P*(*BAG*|*do*(*BMI* = *high*)) is: {low BAG: 0.30, normal BAG 0.38, high BAG: 0.32} (dashed red line). Similarly, differences between conditional probability distribution for observations or interventions are present for smoking and drinking, although they are smaller. For example, the causal effects of being a non-smoker or non-drinker (dashed blue lines) on having delayed brain aging (negative BAG), are slightly smaller than the observational effects (plain blue lines). This suggests that causal structure matters as the observed correlational effects differ from causal effects. While Figure 2 shows probability distribution conditioned on one variable (the cardiovascular risk factor of interest), the model can also be used to estimate probability distribution conditioned on several variables (e.g., the cardiovascular risk factor of interest and sex). Such results are shown in Supplementary material (Supplementary Figures 1, 2), which illustrate that probability distributions differ with sex.

## Discussion

In this work, it was shown that causal structure learning and Bayesian networks enable us to (1) discover causal relationships between the BAG and cardiovascular risk factors and (2) to assess the causal impact of each cardiovascular risk factor on the BAG while accounting for risk factor relationships (i.e., causal effects). Therefore, this work demonstrates the feasibility and advantages of accounting for causal relationships between cardiovascular risk factors, whereas the methods can be easily translated to other clinical problems in neuroimaging.

First, the results demonstrate that causal associations between variables can be discovered by combining a structure learning algorithm and expert knowledge. Overall, the resulting graph shows clinically meaningful and plausible relationships. Data preprocessing steps that were applied, such as the adjustment for age-related bias of the BAG and the use of different threshold values for males and females when discretizing WHR are reflected in the graph as no relationship between BAG and age, and between sex and WHR is present. Other edges such as the effects of sex on all other cardiovascular risk factors, or the effects of all risk factors on BP are in line with general clinical knowledge. The observed effects of sex, WHR, BMI, smoking, and alcohol consumption on the BAG are in line with previous literature in which many various factors were shown to impact the BAG in simple correlation analyses (Cole, 2020; de Lange et al., 2020; Beck et al., 2021; Dinsdale et al., 2021). Mostly linear effects are observed in the graph except for drinking, which has been reported in the past as having non-linear effects on cardiovascular health in general (O’Keefe et al., 2007).

The accuracy of the model’s predictions, evaluated using AUC, are above chance level. This confirms the suitability of the model although most AUC values are low. However, it has to be pointed out that such results are expected due to the small number of variables included in the model and the high loss of information caused by data discretization. Furthermore, the focus of this work is to identify the causal relationship between variables and the feasibility of using such a causal analysis in the context of brain aging biomarkers rather than training an accurate model that can predict BAG from cardiovascular risk factors.

Observing the Bayesian network conditional probability distributions allows us to assess the impact of cardiovascular risk factors conditioned on other factors. On the other hand, intervening on the Bayesian network allows us to investigate the causal effects of cardiovascular risk factors. Therefore, such a model allows us to estimate group-specific probability distributions, when conditioning on different factors, and to account for confounding variables in a systematic and justified way. Indeed, dealing with confounding variables is crucial to correctly assess associations between factors (Alfaro-Almagro et al., 2021). It is necessary to identify the correct confounding variables for a specific task in a first step and then to adjust other variables for them, or to include them as covariates in the model. In studies specifically investigating the effects of cardiovascular risk factors on the BAG, different confounding variables and adjustment methods have been used, making it difficult to compare the findings. For example, Cole (2020) added age, age^2^, sex, height, head size scaling, and mean task fMRI head motion as predictors to their multiple linear regression model, as they found that these variables are correlated with the BAG. de Lange et al. (2020) corrected their cardiovascular risk factors (blood pressure, alcohol intake and stroke risk) for sex, educational level, and ethnic background prior to modeling the relationships between each factor and the BAG, while adding age as a covariate. Dinsdale et al. (2021) removed the following confounding variables from their lifestyle factors and physiological measurements before computing sex-specific correlations between each variable and the BAG: sex, age, head size, head motion, scanner table position, and imaging center. These examples demonstrate discrepancies between studies in terms of the choice of confounding variables and the adjustment method being used. Therefore, the proposed approach provides a unified way to (1) identify variables with true causal relationships using a structure learning algorithm and (2) obtain confounding bias free/causal associations between variables. More generally, this work demonstrates the feasibility and advantages of performing a causal analysis to improve the understanding of causal effects between variables and can be used in many diverse problems.

Although the approach shows promising and plausible first results to study associations between variables, some limitations remain. First, structure learning should be carefully used in conjunction with expert knowledge to validate/correct the graph. Indeed, many different structure learning algorithms exist and can lead to diverging results, thus requiring expert validation (Reisach et al., 2021). The use of discretized data for Bayesian conditional probability fitting results in a loss of information and the chosen thresholds might not be ideal. Thus, more advanced methods for causal inference should be investigated in the future, such as the framework proposed by Pawlowski et al. (2020), which can handle imaging data directly. On the clinical side, limitations related to the data includes the cross-sectional aspect of the study, and the lack of longitudinal information for the cardiovascular risk factors, which were only measured at a single time point. Moreover, the inclusion of more diverse life behavioral and clinical factors into the model would contribute to make it more realistic and to improve its accuracy.

## Conclusion

The present work introduces a proof-of-concept study using tools from causal analysis to improve our understanding of the BAG, which is a promising biomarker for neurodegeneration. The causal relationships identified between the BAG and cardiovascular risk factors illustrate the wide range of biological information captured in the BAG, thus demonstrating its complexity. Moreover, the advantages of the proposed approach can be extended beyond the scope of brain aging for any multivariable analysis comprising confounding variables.

## Data availability statement

The data analyzed in this study is subject to the following licenses/restrictions: The data that support the findings of this study are available from the Study of Health in Pomerania upon reasonable request. Requests to access these datasets should be directed to https://www2.medizin.uni-greifswald.de/cm/fv/ship/.

## Author contributions

PM, MW, JB, and NF designed the study and interpreted the results. PM performed the research, data analysis, and drafted the manuscript. AA performed the data analysis. SL collected the data. All authors revised the manuscript and approved the submitted version.

## References

[B1] Alfaro-Almagro F. McCarthy P. Afyouni S. Andersson J. L. R. Bastiani M. Miller K. L. (2021). Confound modelling in UK Biobank brain imaging. *NeuroImage* 224:117002. 10.1016/j.neuroimage.2020.117002 32502668 PMC7610719

[B2] Ashwell M. (2011). Charts Based on Body Mass Index and Waist-to-Height Ratio to Assess the Health Risks of Obesity: A Review. *Open Obes. J.* 311 78–84. 10.11124/jbisrir-2012-248 27820152

[B3] Baecker L. Garcia-Dias R. Vieira S. Scarpazza C. Mechelli A. (2021). Machine learning for brain age prediction: Introduction to methods and clinical applications. *EBioMedicine* 72:103600. 10.1016/j.ebiom.2021.103600 34614461 PMC8498228

[B4] Beck D. de Lange A.-M. G. Pedersen M. L. Alnæs D. Maximov I. I. Voldsbekk I. (2021). Cardiometabolic risk factors associated with brain age and accelerate brain ageing. *Hum. Brain Mapp.* 43 1–21. 10.1002/hbm.25680 34626047 PMC8720200

[B5] Cole J. H. (2020). Multimodality neuroimaging brain-age in UK biobank: Relationship to biomedical, lifestyle, and cognitive factors. *Neurobiol. Aging* 92 34–42. 10.1016/j.neurobiolaging.2020.03.014 32380363 PMC7280786

[B6] de Lange A.-M. G. Anatürk M. Suri S. Kaufmann T. Cole J. H. Griffanti L. (2020). Multimodal brain-age prediction and cardiovascular risk: The Whitehall II MRI sub-study. *NeuroImage* 222:117292. 10.1016/j.neuroimage.2020.117292 32835819 PMC8121758

[B7] Dinsdale N. K. Bluemke E. Smith S. M. Arya Z. Vidaurre D. Jenkinson M. (2021). Learning patterns of the ageing brain in MRI using deep convolutional networks. *NeuroImage* 224:117401. 10.1016/j.neuroimage.2020.117401 32979523

[B8] Fischl B. Salat D. H. Busa E. Albert M. Dieterich M. Haselgrove C. (2002). Whole Brain Segmentation: Automated Labeling of Neuroanatomical Structures in the Human Brain. *Neuron* 33 341–355. 10.1016/S0896-6273(02)00569-X 11832223

[B9] Franke K. Gaser C. (2019). Ten Years of BrainAGE as a Neuroimaging Biomarker of Brain Aging: What Insights Have We Gained? *Front. Neurol.* 10:789. 10.3389/fneur.2019.00789 31474922 PMC6702897

[B10] Gencoglu O. Gruber M. (2020). Causal Modeling of Twitter Activity during COVID-19. *Computation* 8:85. 10.3390/computation8040085

[B11] Glymour M. Pearl J. Jewell N. P. (2016). *Causal Inference in Statistics: A Primer.* Hoboken: John Wiley & Sons.

[B12] Heinze-Deml C. Maathuis M. H. Meinshausen N. (2018). Causal Structure Learning. *Annu. Rev. Stat. Appl.* 5 371–391. 10.1146/annurev-statistics-031017-100630

[B13] Henschel L. Conjeti S. Estrada S. Diers K. Fischl B. Reuter M. (2020). FastSurfer - A fast and accurate deep learning based neuroimaging pipeline. *NeuroImage* 219:117012. 10.1016/j.neuroimage.2020.117012 32526386 PMC7898243

[B14] Jin Y. Su Y. Zhou X.-H. Huang S. The Alzheimer’s Disease Neuroimaging Initiative (2016). Heterogeneous multimodal biomarkers analysis for Alzheimer’s disease via Bayesian network. *EURASIP J. Bioinforma. Syst. Biol.* 2016:12. 10.1186/s13637-016-0046-9 27610127 PMC4992017

[B15] Khanna S. Domingo-Fernández D. Iyappan A. Emon M. A. Hofmann-Apitius M. Fröhlich H. (2018). Using Multi-Scale Genetic, Neuroimaging and Clinical Data for Predicting Alzheimer’s Disease and Reconstruction of Relevant Biological Mechanisms. *Sci. Rep.* 8:11173. 10.1038/s41598-018-29433-3 30042519 PMC6057884

[B16] Larrañaga P. Moral S. (2011). Probabilistic graphical models in artificial intelligence. *Appl. Soft Comput.* 11 1511–1528. 10.1016/j.asoc.2008.01.003

[B17] Liang H. Zhang F. Niu X. (2019). Investigating systematic bias in brain age estimation with application to post-traumatic stress disorders. *Hum. Brain Mapp.* 40 3143–3152. 10.1002/hbm.24588 30924225 PMC6865701

[B18] Lombardi A. Monaco A. Donvito G. Amoroso N. Bellotti R. Tangaro S. (2021). Brain Age Prediction With Morphological Features Using Deep Neural Networks: Results From Predictive Analytic Competition 2019. *Front. Psychiatry* 11:619629. 10.3389/fpsyt.2020.619629 33551880 PMC7854554

[B19] Mancia G. Fagard R. Narkiewicz K. Redon J. Zanchetti A. Böhm M. (2014). 2013 ESH/ESC Practice Guidelines for the Management of Arterial Hypertension. *Blood Press.* 23 3–16. 10.3109/08037051.2014.868629 24359485

[B20] Marchezini G. F. Lacerda A. M. Pappa G. L. Meira W. Miranda D. Romano-Silva M. A. (2022). Counterfactual inference with latent variable and its application in mental health care. *Data Min. Knowl. Discov.* 36 811–840. 10.1007/s10618-021-00818-9 35125931 PMC8801560

[B21] O’Keefe J. H. Bybee K. A. Lavie C. J. (2007). Alcohol and Cardiovascular Health: The Razor-Sharp Double-Edged Sword. *J. Am. Coll. Cardiol.* 50 1009–1014. 10.1016/j.jacc.2007.04.089 17825708

[B22] Park E. Chang H. Nam H. S. (2018). A Bayesian Network Model for Predicting Post-stroke Outcomes With Available Risk Factors. *Front. Neurol.* 9:699. 10.3389/fneur.2018.00699 30245663 PMC6137617

[B23] Pawlowski N. Castro D. C. Glocker B. (2020). Deep Structural Causal Models for Tractable Counterfactual Inference. *Adv. Neural Inf. Process. Syst.* 33 857–869.

[B24] Pearl J. (2012). The Do-Calculus Revisited. *ArXiv Prepr* [Preprint]. 10.48550/arXiv.1210.4852 35895330

[B25] Reisach A. G. Seiler C. Weichwald S. (2021). Beware of the Simulated DAG! Causal Discovery Benchmarks May Be Easy To Game. *Adv. Neural Inf. Process. Syst.* 34 27772–27784.

[B26] Völzke H. Alte D. Schmidt C. O. Radke D. Lorbeer R. Friedrich N. (2011). Cohort Profile: The Study of Health in Pomerania. *Int. J. Epidemiol.* 40 294–307. 10.1093/ije/dyp394 20167617

[B27] Welcome to CausalNex’s API (2020). *Welcome to CausalNex’s API docs and tutorials! — causalnex 0.11.0 documentation.* Available Online at: https://causalnex.readthedocs.io/en/latest/ (accessed Mar 25, 2022).

[B28] Zheng X. Aragam B. Ravikumar P. K. Xing E. P. (2018). “DAGs with NO TEARS: Continuous Optimization for Structure Learning,” in *Advances in Neural Information Processing Systems*, (San Francisco: Curran Associates, Inc).

