# Supplementary material for: An exploratory causal analysis of the relationships between the brain age gap and cardiovascular risk factors
Source: Front Aging Neurosci. 2022 Aug 22;14:941864. doi: 10.3389/fnagi.2022.941864 (PMC9441743; doi:10.3389/fnagi.2022.941864)
Supplement: Supplementary file 1 [file Data_Sheet_1.pdf]

1.1 Supplementary Figures

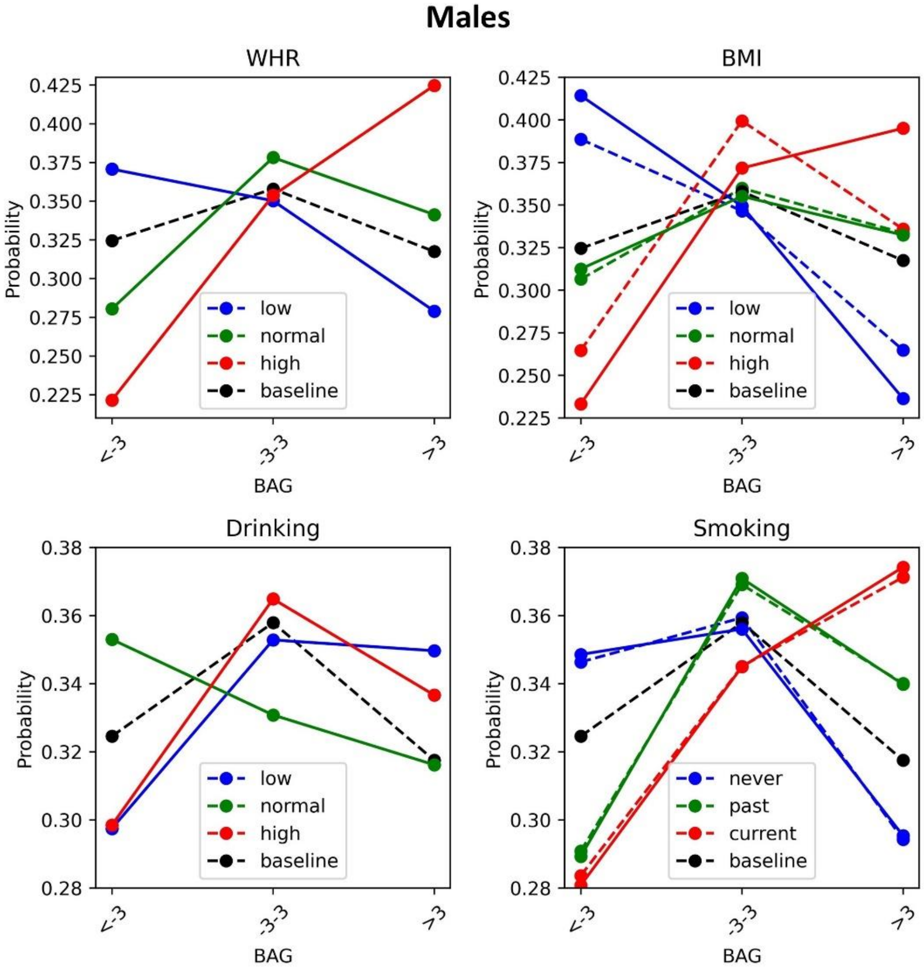

Figure S1. BAG distributions when observing (plain lines) or intervening (dashed lines) on the cardiovascular risk factors, while conditioning on sex (males). BAG: Brain age gap; BMI: Body mass index; WHR: Waist-to-hip ratio.

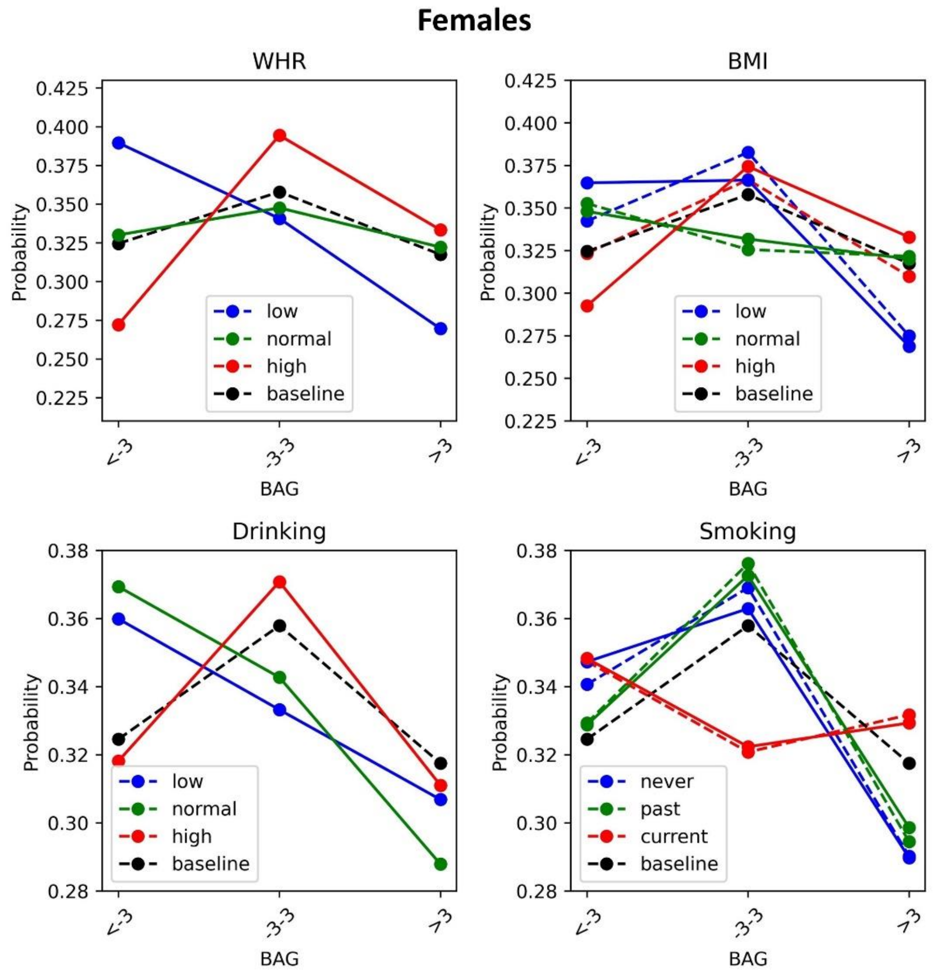

Figure S2. BAG distributions when observing (plain lines) or intervening (dashed lines) on the cardiovascular risk factors, while conditioning on sex (females). BAG: Brain age gap; BMI: Body mass index; WHR: Waist-to-hip ratio.
